# Performance of the international physical activity questionnaire (short form) in subgroups of the Hong Kong chinese population

**DOI:** 10.1186/1479-5868-8-81

**Published:** 2011-08-01

**Authors:** Paul H Lee, YY Yu, Ian McDowell, Gabriel M Leung, TH Lam, Sunita M Stewart

**Affiliations:** 1 FAMILY: A Jockey Club Initiative for a Harmonious Society, School of Public Health, Li Ka Shing Faculty of Medicine, University of Hong Kong, 21 Sassoon Road, Pokfulam, Hong Kong; 2 Department of Epidemiology and Community Medicine, University of Ottawa, 451 Smyth Road, Ottawa, Canada; 3 Department of Psychiatry, University of Texas Southwestern Medical Center at Dallas, 5323 Harry Hines Boulevard, Dallas, Texas, 75390, USA

**Keywords:** Accelerometry, Assessment, Exercise, MET, Validation

## Abstract

**Background:**

The International Physical Activity Questionnaire (IPAQ-SF) has been validated and recommended as an efficient method to assess physical activity, but its validity has not been investigated in different population subgroups. We examined variations in IPAQ validity in the Hong Kong Chinese population by six factors: sex, age, job status, educational level, body mass index (BMI), and visceral fat level (VFL).

**Methods:**

A total of 1,270 adults (aged 42.9 ± SD 14.4 years, 46.1% male) completed the Chinese version of IPAQ (IPAQ-C) and wore an accelerometer (ActiGraph) for four days afterwards. The IPAQ-C and the ActiGraph were compared in terms of estimated Metabolic Equivalent Task minutes per week (MET-min/wk), minutes spent in activity of moderate or vigorous intensity (MVPA), and agreement in the classification of physical activity.

**Results:**

The overall Spearman correlation (ρ) of between the IPAQ-C and ActiGraph was low (0.11 ± 0.03; range in subgroups 0.06-0.24) and was the highest among high VFL participants (0.24 ± 0.05). Difference between self-reported and ActiGraph-derived MET-min/wk (overall 2966 ± 140) was the smallest among participants with tertiary education (1804 ± 208). When physical activity was categorized into over or under 150 min/wk, overall agreement between self-report and accelerometer was 81.3% (± 1.1%; subgroup range: 77.2%-91.4%); agreement was the highest among those who were employed full-time in physically demanding jobs (91.4% ± 2.7%).

**Conclusions:**

Sex, age, job status, educational level, and obesity were found to influence the criterion validity of IPAQ-C, yet none of the subgroups showed good validity (ρ = 0.06 to 0.24). IPAQ-SF validity is questionable in our Chinese population.

## Introduction

Physical activity greatly contributes to overall health and mental well-being and is associated with reduced mortality [1-3], but physical inactivity and sedentary lifestyles have reached epidemic proportions [4]. Much attention has been paid to developing reliable and valid instruments to estimate activity levels and to measure the impact of interventions to promote physical activity [5]. Objective methods for measuring physical activity include motion sensors (e.g., pedometers or accelerometers) and measures of physiological response to exercise, such as heart rate monitors [6,7]. The accelerometer is often used as the gold standard against which self-report questionnaires are compared [8]. Though objective, accelerometers may not always be feasible to use because of cost and inconvenience. A simple and valid self-report measure of physical activity would have the advantages of convenience, rapid data collection and low cost.

Of the many published questionnaires, the International Physical Activity Questionnaire (IPAQ) has been investigated in several populations. The IPAQ was developed by the World Health Organization in 1998 (http://www.ipaq.ki.se) for surveillance of physical activity and to facilitate global comparisons. The 31-item long form and the 9-item short form assess time spent on different activities. The short form records four types of physical activity: vigorous activity such as aerobics; moderate-intensity activity such as leisure cycling; walking, and sitting. The short form is preferred by many researchers because it has equivalent psychometric properties to the long form despite being one-third the length [5]. The two forms have been validated against accelerometer measurements in 12 countries with small samples of 19 to 257 participants. Spearman correlations between the two measurement methods were moderate at best, ranging from -0.12 to 0.57, with a pooled correlation of 0.30 [5]. The IPAQ correlated more closely with an objective measurement of vigorous physical activity than for other activity levels [4]. Despite these variable validity results, a recommendation was made that IPAQ (short form, referring to activity in the past seven days) be used for surveillance and comparison of national trends [4,5].

The modest correlation with objective measurements, combined with the wide variation in reported coefficients, raise concern in universally recommending the IPAQ. Four studies presented sufficient data to allow for more extensive analysis of the agreement between IPAQ and accelerometer readings [9-12]. Using data from these studies, we have calculated that the IPAQ overestimated physical activity compared to accelerometers, by 35% in Switzerland [11], 85% in Vietnam [10], 100% in US [9], and by 170% in Hong Kong [12]. The discrepancy between the measurements, and the wide range of discrepancies, reinforces our concern over the instrument's cross-cultural suitability.

The inconsistent overestimate suggests a bias (albeit to widely varying extents), complicated by random errors in both the IPAQ and accelerometer measurements. One possibility is that the accelerometer is not as reliable as we have believed, although why an ostensibly objective instrument should vary so widely in different settings is not easy to explain. A more plausible explanation is that the IPAQ may be more accurate among some respondent groups than in others, due to differences in translation or group characteristics such as attitudes toward exercise or level of understanding. Given the advantages of IPAQ, including its ease of administration and low cost, it seems worthwhile to investigate whether its validity indices can be improved. A first step may be to test the hypothesis that the instrument performs more adequately in some subgroups than in others. If true, this would imply restriction of its use in groups where it gives valid results, and shed light on how the IPAQ could be corrected or built upon. In this study, we examined variations in IPAQ validity in a sample of Hong Kong Chinese adults, analyzed by subgroups defined in terms of sex, age, job status, educational level, body mass index (BMI), and visceral fat level (VFL).

The translated Chinese version (IPAQ-C) was previously validated in Hong Kong [12] and in Guangzhou [13], with weak-to-moderate correlations with pedometer and accelerometer measurements (ranging from 0.09 [12] to 0.33 [13]). The Guangzhou sample was older than the Hong Kong sample (mean ages 65.2 vs. 28.7) [12,13], so perhaps age may affect the accuracy of IPAQ reporting. Previous studies had also identified sex as a factor that may affect the accuracy of self-reported physical activity [4,14]. Job status may be another factor, since respondents with a regular job may have a routine daily schedule that facilitates recall of their physical activity. The physical demands of the job may also influence reporting accuracy. In addition, educational level may be associated with accuracy of self-reported physical activity data, and it would be expected that there would be a better correlation of IPAQ data with objective measurement among those with more education as they may have a better comprehension of the questions compared to others [5]. Lastly, as overweight people have a different physical activity pattern from others [15] and their self-report could be affected by a social desirability response bias, BMI or visceral fat level (VFL) may also modify the accuracy of self-report data. In this study, we aimed to investigate IPAQ-C accuracy by examining questionnaire-accelerometer correlations by sex, age, job status, educational level, BMI, and VFL.

## Methods

### Participants

This study was part of the Hong Kong Jockey Club FAMILY Project cohort study which includes Hong Kong families recruited since March 2009. Sampling was based on a random selection of residential addresses provided by the Hong Kong Census and Statistics Department. A family was eligible when all members aged 15 years or older, who lived in the same address and could understand Cantonese, agreed to participate. For the present analyses, we used baseline data on the first 5,000 families interviewed during March to October, 2009. All eligible members were interviewed by trained interviewers who entered the data into tablet PCs. Other details of the interview have been described elsewhere [16]. Having completed the survey, participants were invited (all members from the households were invited for half of the households, while for the other half a randomly drawn member was invited) to take part in a sub-study by wearing an accelerometer for four consecutive days (including a weekend). Written consent was obtained from respondents and this study was approved by the Institutional Review Board of The University of Hong Kong.

### Measurements

#### Body composition

Height was measured with SECA 214 stadiometer (http://www.seca.com), with a precision of 1 mm. Weight and VFL was measured with Omron fat analyzer scale HBF-356 (http://www.omron-healthcare.com.sg). Its precision is 0.1 kg for weight and 1 unit for visceral fat level. All measurements were taken in-person by trained interviewers with standard protocols. BMI was calculated by dividing weight (kg) by the square of height (m^2^).

#### IPAQ-C

The 9-item IPAQ-C records self-reported physical activity in the last seven days [12]. Responses were converted to Metabolic Equivalent Task minutes per week (MET-min/wk) [5] according to the IPAQ scoring protocol: total minutes over last seven days spent on vigorous activity, moderate-intensity activity, and walking were multiplied by 8.0, 4.0, and 3.3, respectively, to create MET scores for each activity level. MET scores across the three sub-components were summed to indicate overall physical activity [5].

#### Accelerometer

The ActiGraph is widely used as an objective measurement of physical activity and reported to be reliable and valid [17-19]. The ActiGraph GT1M uni-axial accelerometer (http://www.theactigraph.com) was to be worn around the waist for four consecutive days spanning a weekend for all waking hours, removed only for bathing or sleeping. The choice of the first day (from Thursday, Friday, or Saturday) was up to the participants. Records with less than 600 minutes of registered time in a day were excluded as invalid [4,5].

Following the grouping standard [20], we used one-minute reference period for raw ActiGraph count data. Data (as movement recorded in a one-minute period) were then converted into minutes spent in moderate-intensity (3.00-5.99 METs, 1952-5724 counts per minute) or vigorous activity (≥ 6.00 METs, ≥ 5725 counts per minute) [21]. The MET score per minute (MET-min) for a day was computed with the following formula: 8 × minutes spent in vigorous activity + 4 × minutes spent in moderate-intensity activity. As the IPAQ covered 7 days but the ActiGraph only covered 4 days (including a weekend), we averaged the 4-day ActiGraph data according to the day of the week, and obtained a weekly MET-min score by 5 × average weekday MET-min + 2 × average weekend MET-min.

#### Other measurements

In addition to the IPAQ, the interview obtained demographic information and questions related to psychosocial functioning. Tertiary education refers to those with a bachelor's degree or further education.

### Statistical Analysis

Outliers on ActiGraph scores (> median + 1.5 inter-quartile range) and missing IPAQ-C data were removed from the analysis. Independent *t*-tests were used to compare the differences in the amount of moderate-intensity, vigorous, and total physical activity between IPAQ-C and ActiGraph. Because the MET-min/wk measurements of neither the IPAQ-C nor ActiGraph were normally distributed, Spearman correlations were used to determine the correlations between IPAQ-C and ActiGraph records (minutes and count data) by activity level [5]. The Fisher's *r *to *z*-test was used to compare the difference between pairs of correlations. Correlations and differences are presented with standard error for computation of confidence interval as appropriate. ActiGraph-min equals 2 × minutes spent in vigorous activity + minutes spent in moderate-intensity activity, and ActiGraph-count equals raw counts in hours with any movement. The proportions of respondents who met the Centers for Disease Control - American College of Sports Medicine (CDC-ACSM) guideline, i.e., moderate-intensity min/wk + 2 × vigorous min/wk ≥ 150 [22], were computed with both the IPAQ-C and ActiGraph data. We assessed the agreement between the two proportions by comparing the observed proportion with the same classification to the percent agreement that could have occurred by chance. To further examine the agreement of CDC-ACSM classification between IPAQ-C and ActiGraph, we categorized respondents into equal-sized groups according to IPAQ and ActiGraph records, and reported the proportion classified in the same group by both methods. The observed proportions were also compared to chance agreement (for 2 groups: probability of being classified in the same activity group by both methods; for 3 groups: 33.3%; for 4 groups: 25.0%). In addition, the ActiGraph-measured MET-min/wk was compared across IPAQ categories with one-way ANOVA. ANOVA results with significant *P*-values (< 0.05) were further analyzed with the Tukey's method. All statistical analysis was performed using Predictive Analytics SoftWare (PASW 18.0, formerly known as SPSS).

## Results

Out of 11,713 respondents from 5,000 families, 2,511 (21.5%) respondents wore the ActiGraph. The characteristics of ActiGraph wearers and non-wearers were comparable, except for age (wearers 42.9 years, vs 44.8 for non-wearers, *P *< 0.001), job status (58.7% full-time employment for wearers vs 49.4% for non-wearers, *P *< 0.001), and percentage of respondents passing the CDC-ACSM guideline (passing rate = 92.5% for wearers vs 47.1% for non-wearers). Excluding ActiGraph invalid data (either wearing for less than four days or not following the 2 weekdays + 2 weekends format) (n = 1,151) and IPAQ missing data (n = 90), we kept 1,270 respondents in the present analysis: 10.8% of the whole sample. There were no significant differences between the characteristics of the valid and invalid samples.

Table 1 shows that 585 (46.1%) of the respondents were male, 735 (58.3%) had a full-time job, 299 (24.3%) attained tertiary education, and 399 (31.5%) were overweight based on BMI (≥ 25), or 347 (29.5%) overweight based on VFL (≥ 10%). The mean age was 42.9 years (range: 15 to 82 years, inter-quartile range = 20 years).

**Table 1:** Demographic characteristics of the 1,270 respondents.

		n	Age mean (S.D.)	Male n (row %)	Full-time worker n (row %)	Tertiary Education n (row %)	Weight (kg) mean (S.D.)	Height (cm) mean (S.D.)	BMI mean (S.D.)	VFL mean (S.D.)
Total		1270	42.9 (14.4)	585 (46.1%)	745 (58.7%)	299 (24.3%)	61.6 (12.4)	161.8 (8.7)	23.5 (3.9)	7.5 (4.6)

Sex										
	Male	585	43.5 (15.3)	N/A	376 (64.3%)	153 (26.9%)	64.9 (12.5)	165.5 (7.9)	23.6 (3.8)	8.7 (4.9)
	Female	685	42.4 (13.5)	N/A	369 (53.9%)	146 (22.0%)	58.8 (11.7)	158.5 (8.1)	23.4 (4.0)	6.5 (4.2)
Age, years										
	≤29	232	21.7 (4.3)	110 (47.4%)	98 (42.2%)	85 (38.0%)	58.9 (13.9)	164.2 (9.4)	21.7 (4.0)	4.4 (3.5)
	30-49	629	40.3 (5.6)	273 (43.4%)	472 (75.0%)	172 (28.0%)	62.9 (12.6)	162.3 (8.8)	23.8 (3.9)	7.3 (4.4)
	≥ 50	409	59.0 (7.7)	202 (49.4%)	175 (57.2%)	42 (10.7%)	61.2 (11.0)	159.5 (7.8)	24.0 (3.6)	9.2 (4.7)
Full-time worker										
	Yes - high PD	105	44.5 (9.9)	61 (58.1%)	N/A	7 (6.9%)	65.3 (12.5)	163.7 (8.4)	24.3 (3.7)	8.7 (5.0)
	Yes - low PD	630	41.1 (10.3)	310 (49.2%)	N/A	211 (34.5%)	62.5 (12.4)	163.1 (8.7)	23.4 (3.7)	7.3 (4.5)
	Not full-time	525	54.1 (8.8)	303 (46.0%)	350 (53.1%)	87 (13.6%)	62.4 (11.5)	160.2 (8.3)	24.2 (3.7)	8.9 (4.7)
Tertiary education										
	Yes	299	37.2 (12.0)	153 (51.2%)	224 (74.9%)	N/A	62.2 (12.8)	163.8 (9.0)	23.1 (3.7)	6.8 (4.7)
	No	933	44.7 (14.5)	416 (44.6%)	499 (53.5%)	N/A	61.4 (12.1)	160.9 (8.7)	23.7 (3.9)	7.7 (4.6)
BMI										
	Overweight (≥ 25)	399	46.1 (12.4)	201 (50.4%)	317 (60.7%)	84 (21.8%)	73.9 (10.7)	162.4 (9.1)	28.0 (2.7)	12.3 (3.9)
	Normal(< 25)	868	41.2 (15.0)	382 (44.0%)	501 (57.7%)	214 (25.4%)	55.9 (8.4)	161.5 (8.5)	21.4 (2.3)	5.2 (2.9)
VFL, %										
	Overweight (≥ 10)	347	49.9 (12.1)	219 (63.1%)	211 (60.8%)	75 (22.4%)	74.0 (10.5)	164.0 (8.8)	27.5 (3.1)	13.4 (3.2)
	Normal(< 10)	830	41.4 (12.9)	316 (38.1%)	515 (62.1%)	214 (26.6%)	56.7 (9.1)	160.6 (8.5)	22.0 (2.8)	5.0 (2.4)

PD: physical demand, BMI: body mass index, VFL: visceral fat level

Table 2 shows that self-reported MET-min per week exceeded the ActiGraph readings by 231% for total physical activity, by 236% for moderate-intensity, and by 1047% for vigorous-intensity physical activity (*P *< 0.001 for all comparisons). Although physical activity time reported in IPAQ-C was significantly greater than that measured by ActiGraph, the two measurements were positively correlated (Table 2). The correlation between IPAQ-C and ActiGraph MET-min was significant but weak for total physical activity, moderate-intensity activity, as well as for vigorous-intensity activity. The correlations between ActiGraph count data and IPAQ-C moderate min, IPAQ-C vigorous min, IPAQ-C MET-min were significant but also weak. As reported in previous research [23], the IPAQ-ActiGraph correlation was higher when results were expressed in counts than in total MET-min (ρ = 0.16 vs 0.11, *P *< 0.05).

**Table 2:** Comparisons of IPAQ-C and ActiGraph for three categories of physical activity.

	Moderate activity Minutes per day	Vigorous activity Minutes per day	Total MET Per week
IPAQ-C, mean(SD)	146.2 (164.8)	13.2 (46.1)	4250.6 (5053.9)
ActiGraph, mean(SD)	43.6 (23.9)	1.2 (3.1)	1284.3 (728.1)
IPAQ-C vs ActiGraph, difference (SE)	102.6*** (4.6)	12.0*** (1.3)	2966.3*** (140.1)
IPAQ-C vs ActiGraph -min, Spearman ρ correlation (SE)	0.09** (0.03)	0.16*** (0.03)	0.11*** (0.03)
IPAQ-C vs ActiGraph-count, Spearman ρ correlation (SE)	0.14*** (0.03)	0.06* (0.03)	0.16*** (0.03)

MET: metabolic equivalent task per week (ActiGraph: 8*vigorous min + 4*moderate min, IPAQ: 8*vigorous min + 4*moderate min + 3.3*walking min)

* *P *< 0.05.

** *P *< 0.01.

*** *P *< 0.001.

Table 3 further shows that, in general, the correlations between IPAQ-reported MET and ActiGraph were higher when ActiGraph raw count data were used. In terms of IPAQ total MET by subgroup, IPAQ-ActiGraph correlations appeared to be higher for males, older age groups, those with a full-time job of high physical demand, those with lower education attainment, and those who were overweight (by classification of either BMI or VFL), yet none of these effects reached a significant level except VFL (*P *= 0.01). The highest correlation between IPAQ total MET and ActiGraph was found among those with higher VFL (ActiGraph count data, ρ = 0.31). Furthermore, the IPAQ-ActiGraph correlation was higher among those with higher VFL than those with normal VFL, regardless of physical activity groups or the ActiGraph measurements used. In contrast, the lowest correlation between IPAQ total MET and ActiGraph was found among those aged 29 years or younger (ActiGraph count data, ρ = 0.04).

**Table 3:** Spearman correlations of IPAQ-C and ActiGraph-measured physical activity by subgroup using ActiGraph time and count data

		ActiGraph activity levels and measurements			
		
		ActiGraph-min (moderate min)	ActiGraph-min (vigorous min)	ActiGraph-min	ActiGraph-count
**Respondent characteristics\IPAQ activity level**		**Moderate**	**Vigorous**	**Total MET**	**Total MET**

Sex					
	Male	0.10*	0.23***	0.14***	0.18***
	Female	0.09*	0.09*	0.09*	0.15***
Age, years					
	≤29	0.05	0.21***	0.06	0.04
	30-49	0.09*	0.12**	0.12**	0.19***
	≥ 50	0.12*	0.14**	0.15**	0.25***
Full-time worker					
	Yes - high PD	0.19*	0.25**	0.18*	0.16
	Yes - low PD	0.10*	0.10**	0.12**	0.20***
	Not full-time	0.06	0.21***	0.07	0.08
Tertiary education					
	Yes	0.03	0.17**	0.09	0.08
	No	0.11***	0.17***	0.12***	0.18***
BMI					
	Overweight (≥ 25)	0.10	0.22***	0.14**	0.21***
	Normal (< 25)	0.09**	0.14***	0.10**	0.14***
VFL, %					
	Overweight (≥ 10)	0.18***	0.23***	0.24***	0.31***
	Normal (< 10)	0.08*	0.10**	0.09*	0.14***

ActiGraph-min: time spent in moderate-to-vigorous physical activity (2 × minutes spent in vigorous activity + minutes spent in moderate-intensity activity, ActiGraph-count: raw counts in hours with any movement recorded, MET: metabolic equivalent task per week (8*vigorous min + 4*moderate min + 3.3*walking min), PD: physical demand

* *P *< 0.05.

** *P *< 0.01.

*** *P *< 0.001.

Table 3 also shows the IPAQ-ActiGraph correlations for physical activity subgroups classified by both IPAQ report and ActiGraph data (only in MET-min). Regarding moderate-intensity activity, the correlations had similar patterns as those found with total MET. However, for the vigorous activity level, the patterns of the correlations were inconsistent by age or employment group.

Table 4 compares total time spent on physical activity reported in the IPAQ-C to ActiGraph readings, by subgroup. On every comparison, the self-report questionnaire produced much higher estimates of time spent on physical activity than the objective device (by 151% to 5670%). However, the overestimates were not consistent across groups. For time spent on moderate-intensity activity, men overestimated slightly less than women did (differences in min/day = 92.4 vs 111.3, *P *< 0.05), but on vigorous activity men overestimated more (min/day = 16.1 vs 8.5, *P *< 0.01). The comparisons across groupings by body mass (lack of statistical significance) or visceral fat (*P *< 0.05) had a similar reverse pattern regarding time spent on different levels of physical activity. Those with physically demanding full-time jobs overestimated their physical activity time to a greater extent compared to others, approximately two times more on moderate-intensity activity and seven times more on vigorous activity (*P *< 0.001). Those with tertiary education overestimated their exercise time to a lesser extent than respondents without (*P *< 0.001). There was no observable pattern of overestimation by age group, although younger people seemed to have overestimated to a greater extent compared to those aged 30 or over.

**Table 4:** Average time (in minutes per day) spent on physical activity measured by the IPAQ-C and ActiGraph, and differences between the two measurements, by level of activity and respondent characteristics

		Moderate intensity activity per day			Vigorous intensity activity per day			Metabolic equivalent task per week✩		
		IPAQ-C#	ActiGraph#	Difference†	IPAQ-C#	ActiGraph#	Difference†	IPAQ-C#	ActiGraph#	Difference†
Sex										
	Male	137.7 (147.5)	45.2 (23.9)	92.4*** (6.1)	17.4 (51.5)	1.3 (3.4)	16.1*** (2.1)	4290.5 (5124.8)	1339.6 (736.7)	2950.9*** (209.9)
	Female	153.4 (178.0)	42.1 (23.9)	111.3*** (6.7)	9.6 (40.7)	1.0 (2.8)	8.5*** (1.6)	4216.6 (4996.1)	1237.1 (717.8)	2979.5*** (188.2)
Age, years										
	≤29	150.0 (157.0)	39.1 (20.8)	110.9*** (10.3)	17.7 (39.9)	1.4 (3.2)	16.3*** (2.6)	4556.9 (4674.1)	1171.2 (635.7)	3385.8*** (305.8)
	30-49	143.6 (173.5)	43.8 (22.3)	99.8*** (6.9)	11.8 (45.0)	1.00 (2.7)	10.8*** (1.8)	4118.9 (5139.1)	1282.2 (679.5)	2836.7*** (203.0)
	≥ 50	147.9 (155.4)	45.7 (27.4)	102.2*** (7.5)	12.8 (50.9)	1.3 (3.6)	11.5*** (2.5)	4279.5 (5132.5)	1351.8 (835.2)	2927.7*** (248.6)
Full-time worker										
	Yes - high PD	253.9 (233.2)	57.7 (33.2)	196.2*** (22.1)	57.7 (33.2)	1.0 (2.6)	56.3*** (10.6)	9384.4 (8721.6)	1671.8 (966.3)	7712.6*** (835.3)
	Yes - low PD	142.5 (174.2)	44.3 (21.7)	98.2*** (6.9)	8.7 (32.9)	1.0 (2.8)	7.7*** (1.3)	3904.9 (4834.3)	1297.4 (664.7)	2607.6*** (191.1)
	Not full-time	130.2 (125.6)	40.1 (23.2)	90.1*** (5.6)	10.0 (32.9)	1.3 (3.5)	8.6*** (1.4)	3679.3 (3580.6)	1196.2 (723.3)	2483.2*** (158.6)
Tertiary education										
	Yes	107.0 (126.0)	42.7 (20.0)	64.2*** (7.4)	9.0 (22.6)	1.1 (2.9)	7.9*** (1.3)	3062.4 (3595.4)	1258.6 (599.1)	1803.9*** (208.4)
	No	157.8 (173.8)	43.7 (25.1)	114.1*** (5.6)	14.4 (51.5)	1.2 (3.2)	13.2*** (1.7)	4601.9 (5386.8)	1290.3 (766.8)	3311.7*** (174.0)
BMI										
	Overweight (≥ 25)	152.9 (174.1)	44.5 (24.6)	108.4*** (8.6)	13.0 (42.2)	1.2 (3.4)	11.8*** (2.1)	4411.7 (5339.2)	1315.0 (771.6)	3096.7*** (263.5)
	Normal (< 25)	143.4 (160.5)	43.1 (23.6)	100.3*** (5.4)	13.3 (47.9)	1.1 (2.9)	12.2*** (1.6)	4187.3 (4923.9)	1270.5 (706.4)	2916.8*** (165.4)
VFL, %										
	Overweight (≥ 10)	134.9 (150.4)	47.9 (25.9)	87.1*** (7.9)	14.6 (51.2)	1.4 (3.7)	13.2*** (2.8)	4063.8 (5231.5)	1416.8 (811.1)	2647.0*** (274.5)
	Normal (< 10)	150.2 (170.2)	42.6 (23.2)	107.6*** (5.8)	11.8 (44.7)	1.0 (2.7)	10.8*** (1.6)	4271.6 (5044.0)	1251.4 (693.5)	3020.2*** (172.9)

PD: physical demand

✩ 8*vigorous min + 4*moderate min, IPAQ: 8*vigorous min + 4*moderate min + 3.3*walking min

# Data are presented as mean (standard deviation).

† Data are presented as mean (standard error).

* *P *< 0.05.

** *P *< 0.01.

*** *P *< 0.001.

We assessed the agreement of the two measurements in classifying respondents in terms of meeting the CDC-ACSM physical activity guideline (details can be found in Additional file 1). We found that the overall IPAQ-ActiGraph agreement was only slightly better than chance agreement (81.3% vs 79.6%, *P *< 0.001). The agreement in the classification was better among respondents who had a physically demanding full-time job than those with physically non-demanding full-time jobs and those without full-time jobs (91.4%, 82.5%, and 77.9%, respectively, *P *< 0.05). Males had higher agreement between the two classifications than did females (83.9% vs 79.0%, *P *< 0.05).

We also assessed the IPAQ-ActiGraph agreement in classifying respondents into tertile and quartile of activity level, against classification based on chance (33% for tertile and 25% for quartile). The observed agreement was significantly better than chance except for the group aged ≤29 years and those with tertiary education.

**Figure 1:**
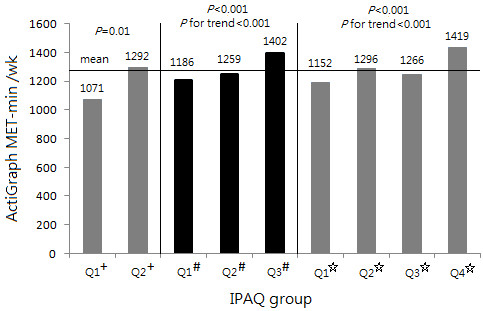
**ActiGraph-measured time on physical activity (MET min/wk) by equal groups of IPAQ-C**. + Q1: time spent in moderate-to-vigorous physical activity < 150 minutes per week; Q2: time spent in moderate-to-vigorous physical activity ≥ 150 minutes per week. # Q1, Q2, and Q3 are the first, second, and third tertile, respectively. ✩ Q1, Q2, Q3, and Q4 are the first, second, third, and fourth quartile, respectively.

Lastly, we compared the mean MET min/wk measured by ActiGraph across equal-sized groups based on IPAQ scores (Figure 1). Overall, the ActiGraph readings were higher for groups classified by IPAQ as being more active than for less active groups. The mean ActiGraph-measured time was significantly different by IPAQ grouping in all three groupings (*P *< 0.001). In the 3-group comparison, the ActiGraph MET min/wk in the highest IPAQ group was significantly more than the other two groups (1186 vs 1402, *P *< 0.001; 1259 vs 1402, *P *< 0.05, respectively), but the difference in MET min/wk between the other two groups was not significant (1186 vs 1259, *P *= 0.31). In the 4-group comparison, the ActiGraph MET min/wk in the highest IPAQ group (group 4) was significantly more than groups 1 and 3 (1152 vs 1419, *P *< 0.001; 1266 vs 1419, *P *< 0.05, respectively), but the differences among the other three groups were not significant (1152 vs 1296 vs 1266, *P *= 0.06, *P *for trend = 0.28).

## Discussion

Although the IPAQ has been recommended as a surveillance instrument, we argue that the validation studies of IPAQ do not generally provide strong empirical support for its validity compared against objective measures of physical activity [4,5,12,13,23,24]. The correlations of 0.30 [5] are far lower than the agreement between self-report and objective measurements of other health variables, such as smoking [25], body weight [26] or hypertension [27]. To rule out Simpson's paradox [28] (i.e., signs of correlation are positive in all groups, but the correlation becomes negative when groups are pooled together), we studied correlations of the IPAQ with an objective measurement in different subgroups. This would also indicate whether the questionnaire instrument works better for certain subgroups. To our knowledge, this was the first study to examine how demographic factors and obesity affect the correlation, difference, and agreement between IPAQ and ActiGraph measurements. However, none of the subgroups showed an acceptable IPAQ-ActiGraph correlation, although the correlations did seem to be higher in certain groups (e.g. males and those with high VFL). The Spearman correlations for all groups in this study were positive, but lay at the lower end of the range of previously reported figures (-0.12 to 0.57) [5,29]. Based on our findings, we question the validity of IPAQ-SF when administered to Hong Kong Chinese respondents.

Contrary to our expectation, differences in age, work-related physical activity level, education, and BMI did not appear to influence the correlation between IPAQ and ActiGraph. Regarding the slightly higher correlation among those with higher VFL, we postulated that perhaps they were more conscious of their physical activities. In support of this, we found that respondents with higher VFL had higher variation in ActiGraph-measured total physical activity (sd = 811.1 vs 693.5 for lower VFL group, *P *< 0.001), which may mean they had a more distinctive physical activity pattern, hence easier to recall. The strength of the IPAQ-ActiGraph correlation was weak among those did not have tertiary education and weaker for those did (Table 3). However, there was no statistical significance when the two correlations were compared (*P *> 0.05). On the other hand, in considering absolute differences between the two methods of measurement (Table 4), over-reporting by respondents without tertiary education nearly doubled that of those with tertiary education (differences in MET-min/wk: 3317.7 vs 1803.9, *P *< 0.001). The performance of the IPAQ is better among those with higher education.

Although over-reporting with activity questionnaires is ubiquitous and has been linked to social desirability bias [30], there were several possible explanations why the correlations in our study were lower than those previously reported. First, we asked the respondents to wear the activity monitor *after *they had completed the IPAQ, while in other studies respondents were often asked to wear the device before they took the IPAQ. The latter approach could have yielded higher IPAQ-ActiGraph agreement, as the self-report responses may have been modified because of the increased awareness arising from wearing the activity monitor. Also, in our study the IPAQ recall period preceded the time when the ActiGraph was worn by one to two weeks. This different time-period could have contributed to the lower correlation (0.16) compared to studies that used the same time-period (0.30) [5]. However, given the stability of IPAQ (3- to 7-day test-retest reliability: 0.81 [5]), we do not believe that having the same recall periods would have substantially altered the results.

Second, the IPAQ has been found to overestimate physical activity to a greater extent than other physical activity questionnaires, such as the Active Australia Survey and the U.S. Behavioral Risk Factor Surveillance System [24]. In this study, the IPAQ overestimated physical activity measured by the ActiGraph from 149% to 461% (mean 231%), which was similar to the finding previously reported in Hong Kong (173%) [12].

Third, how the ActiGraph was applied in different studies may have led to the differences in results. In this study, the respondents were instructed to remove the ActiGraph during aquatic activities because it is not waterproof. Therefore, movement during activities such as swimming would not have been captured. Second, respondents were instructed to wear the ActiGraph on the hip, as suggested in Trost *et al*. [31]. Thus, the ActiGraph may not have accurately measured physical activity during which movement of the hip was limited, such as cycling. It has been reported that Hong Kong young adults swim and ride bicycles more often than older adults [32]. Because accelerometers underestimate these activities, this could be an explanation for our finding of weak IPAQ-ActiGraph correlation in young adults. Furthermore, In a Hong Kong survey, swimming and cycling was the favorite sports activity for 11% and 6%, respectively, of the respondents [33]. Thus, the underestimation of ActiGraph-measured physical activity may not have been negligible in this study. In sum, these three sources together may probably have had an effect on reducing the IPAQ-ActiGraph correlation in this study.

In practice, physical activity measurements may be most relevant in grouping participants into different intensity levels of physical activity (e.g., into two or three groups). The conventional classification scheme is ≥ 150 minutes per week of physical activity of at least moderate intensity [5,22,24]. Based on this guideline, classification of activity by IPAQ and ActiGraph agreed closely (81.3%), although barely better than what could have been achieved by chance (79.6%). Furthermore, regardless of how the respondents were grouped, the IPAQ-ActiGraph agreements were only slightly better than by-chance agreement.

The IPAQ-ActiGraph agreement in classification was slightly better than a chance agreement, but the two measurements did seem to correlate better among those who were more physically active. There was a linear trend in ActiGraph-measured time when we grouped the respondents into three equal-sized groups by IPAQ. However, when the respondents were divided into four IPAQ groups, the intermediate groups were not clearly different in terms of their objectively-measured activity levels. This agrees with a previous finding in Japan [34] that showed IPAQ could only roughly classify mildly and moderately active respondents.

Our results provided some insights for possible modifications of IPAQ-C. First, job-related physical activity level seemed to have had an effect on the difference between IPAQ and ActiGraph measurements. Those who performed in highly physically demanding conditions had the largest difference between their self-report and the ActiGraph-measured physical activity. In particular, they reported an average of 57.7 minutes of vigorous physical activity per day, which was over six times that of the self-reported vigorous physical activity by the other respondents. However, according to the ActiGraph on average they only did 1.0 minute of vigorous physical activity per day, no more than the vigorous physical activity performed by other respondents. Conceivably, however, the Actigraph under-estimated lifting activities. This raises the possibility that the accuracy of the IPAQ-C may be improved by separating physical activity into occupational and leisure types (as in the Global Physical Activity Questionnaire) [35]. Because respondents overestimated occupational physical activity more than other types of activity, reducing the weight of occupational activity may improve the accuracy of IPAQ total MET score. Furthermore, separating physical activity into occupational and leisure types could allow researchers to analyze the benefits of physical activity, at work and at leisure, in relation to health [36].

Second, more detailed instructions [37] may be needed. For those with lower education, more concrete examples of different levels of physical activity intensity may be necessary, as our results indicated that this group had exaggerated their total physical activity more than the others.

The study had several limitations. First, those who agreed to wear the accelerometer might have been healthy volunteers, with different physical activity patterns from those who were less active, as the percentage of respondents who passed the CDC-ACSM guideline was double that of non-respondents. Also, those who were extremely active might have found it too much of a burden to wear the accelerometer and declined to participate. Nevertheless, the results indicated that, demographically, those who wore the accelerometer were not different from the rest of the sample except for being slightly younger and less likely to have full-time employment. Second, although the accelerometer has been used as gold standard for questionnaire validation [17-19], we did not have evidence for its validity or reliability in this study. Lastly, similar to other IPAQ validation studies, we adopted the cut-off points for intensity level suggested by Freedson *et al*. [21], which have not been validated in Chinese populations [12]. However, given our consistent results with the different classification schemes, we do not expect that different cut-off values would yield significantly different findings.

## Conclusions

Although the IPAQ has been recommended and widely used, it has not been found to correlate highly with objective measurements of physical activity, and tends to overestimate MET scores. We investigated the criterion validity of the IPAQ in a Hong Kong Chinese population, grouping our sample by several different variables. We found that it performed poorly in most subgroups when compared to accelerometer data, but slightly better for the highly active respondents.

Despite such low correlations of the IPAQ with ActiGraph in the Chinese population, it is one of the easiest of physical activity questionnaires to administer with less than 10 questions [38]. A correlation of 0.3 - 0.4 is perhaps as close as can be expected for criterion validity of a physical activity questionnaire with 10 questions, against a mechanical device that detects body movement. Further research to improve IPAQ is urgently needed.

## Competing interests

The authors declare that they have no competing interests.

## Authors' contributions

All authors contributed substantially to the design, implementation, analysis and writing of the present paper. The project was designed by GML and THL, the data collection was performed by PHL and YYY, the analysis of the data and interpretation was conducted by PHL, YYY, IM, THL, and SMS the paper was drafted by PHL with significant revision by YYY, IM, GML, THL, and SMS. All authors read and approved the final manuscript.

## Supplementary Material

Additional file 1 Agreement between IPAQ-C and ActiGraph classification by CDC-ACSM physical activity guideline Click here for file
