# Supplementary material for: Performance of the international physical activity questionnaire (short form) in subgroups of the Hong Kong chinese population
Source: Int J Behav Nutr Phys Act. 2011 Aug 1;8:81. doi: 10.1186/1479-5868-8-81 (PMC3157408; doi:10.1186/1479-5868-8-81)
Supplement: Additional file 1 — Agreement between IPAQ-C and ActiGraph classification by CDC-ACSM physical activity guideline [file 1479-5868-8-81-S1.DOC]

Additional File 1. Agreement between IPAQ-C and ActiGraph classification by CDC-ACSM# physical activity guideline

|  |  | By activity guideline | | | | By 3 or 4 equal-size categories | | | |
| --- | --- | --- | --- | --- | --- | --- | --- | --- | --- |
|  |  | % meeting guideline | | Agreement (%) | Agreement  by chance (%) | Agreement (%) by ActiGraph-min | | Agreement (%) by ActiGraph-count | |
|  |  | IPAQ-C | ActiGraph |  |  | (3 categories) | (4 categories) | (3 categories) | (4 categories) |
| Sex* |  |  |  |  |  |  |  |  |  |
|  | Male | 93.0 | 87.9 | 83.9+ | 82.6 | 38.3+++ | 27.9+++ | 40.3+++ | 29.9+++ |
|  | Female | 92.1 | 82.2 | 79.0++ | 77.1 | 35.2+ | 27.2++ | 35.3+ | 29.9+++ |
| Age, years*† |  |  |  |  |  |  |  |  |  |
|  | ≤29 | 93.1 | 78.1 | 77.2+ | 74.2 | 34.9 | 27.2 | 31.5 | 26.3 |
|  | 30-49 | 91.9 | 87.9 | 83.0+ | 81.8 | 37.0+++ | 26.9++ | 40.0+++ | 28.6+++ |
|  | ≥50 | 93.2 | 84.0 | 80.9+ | 79.2 | 37.7+++ | 30.9+++ | 38.5+++ | 34.6+++ |
| Full-time worker*☆† | |  |  |  |  |  |  |  |  |
|  | Yes- high PD | 96.2 | 93.3 | 91.4 | 90.0 | 42.8+++ | 41.0+++ | 47.6+++ | 41.9+++ |
|  | Yes – low PD | 92.1 | 88.9 | 82.5 | 82.7 | 36.4+++ | 27.0++ | 37.8+++ | 28.7+++ |
|  | Not full-time | 92.4 | 78.3 | 77.9+++ | 74.0 | 35.8++ | 25.7 | 35.8+++ | 29.1+++ |
| Tertiary education† | |  |  |  |  |  |  |  |  |
|  | Yes | 91.0 | 85.6 | 79.9 | 79.2 | 32.4 | 24.4 | 33.1 | 24.1 |
|  | No | 92.8 | 84.4 | 81.5+++ | 79.4 | 38.2+++ | 28.4+++ | 39.4+++ | 31.9+++ |
| BMI† |  |  |  |  |  |  |  |  |  |
|  | Overweight (≥25) | 93.0 | 82.7 | 78.2 | 78.1 | 38.9+++ | 27.8++ | 41.6+++ | 30.6+++ |
|  | Normal (<25) | 92.4 | 85.8 | 82.6+++ | 80.3 | 35.6++ | 27.3+++ | 35.8+++ | 29.7+++ |
| VFL, % |  |  |  |  |  |  |  |  |  |
|  | Overweight (≥10) | 93.4 | 86.2 | 82.4 | 81.4 | 38.3+++ | 28.5+++ | 42.1+++ | 31.4+++ |
|  | Normal (<10) | 91.9 | 85.3 | 81.1++ | 79.6 | 36.5+++ | 27.8+++ | 36.9+++ | 29.9+++ |
| Total |  | 92.5 | 84.8 | 81.3+++ | 79.6 | 36.6+++ | 27.5+++ | 37.6+++ | 29.9+++ |

PD: physical demand

* Significantly different on agreement of classification by activity guideline at 5% level.

+, ++, +++ Significantly different from agreement by chance at 5%, 1%, and 0.1% level respectively.

☆ Significantly different on agreement of classification by ActiGraph-min categories at 5% level.

† Significantly different on agreement of classification by ActiGraph-count categories at 5% level.

# CDC-ACSM: Centers for Disease Control – American College of Sports Medicine
